# Cardiac Hypertrophy and Dilatation

**Published:** 1915-04

**Authors:** M. T. Davis

**Affiliations:** Atlanta, Ga.


### CARDIAC HYPERTROPHY AND DILATATION.

By M. T. Davis, M. D., Atlanta, Ga.

Hypertrophy and Dilatation are not two separate processes, but the component halves of one process—nature's conservative effort to afford more power to the heart, which results in the development of new fibres identical with the normal fibres. It is a true hyperplasia.

Functional activity of a muscle favors its development or its atrophy, according as the general powers of nutrition are good or feeble.

Increased demand upon the heart causes palpitation; if persistent, hypertrophy results, provided the nutritive powers are good; if they are defective, dilatation, either pure and simple, or blended in some degree with hypertrophy, is the consequence.

Dilatation is a permanent condition of distention, with imperfect emptying of the ventricle. The sense of this distention is received by the sensory nerves, and carried to the cardiac ganglia, resulting in more frequent and more energetic discharges—a species of palpitation. When this is persistent, hyperplasia of the muscle fibres follows from the dilatation of the blood vessels of the heart, and the increased nutrition, the vessels dilating in response to the well-known law that they so act in a muscle put in motion by irritating its motor nerve.

The two conditions causing increased demand upon the heart, and thus leading to hypertrophy, dilatation, or, more commonly, to both combined, are (1) obstruction to the flow of blood out of the ventricles, which consequently are imperfectly emptied. (2) increased internal pressure from an abnormally powerful distending force, since the column of blood entering the heart finds it already partially filled. Thus the ventricle is soon over-distended, and rapid and powerful contractions follow the heart's effort to empty itself. If not quite equal to the task, excited contractions, that form of palpitation, the evidence of muscular inability results, which, if persistent, leads, by dilatation of the coronary vessels and increased nutrition, to Hypertrophy, or to Dilatation, according to the general nutritive powers of the patient.

Among the obstructions leading to trophic changes in the heart, may be mentioned aortic stenosis, pressure of tumors on the aorta, distortion of the spine by Rickets, violent sustained efforts causing contraction of the muscles in their sheath, with compression of the arteries, and, above all, arterio-sclerosis, interfering with the flow out of the arteries. All these make increased demands upon the propelling power of the heart.

In considering these obstructive causes, let us not forget that increased distending force is a potent factor in Hypertrophy. I shall endeavor to prove this by asking you to consider with me the hypertrophy of the left ventricle, which follows aortic regurgitation and which is also commonly found with mitral regurgitation. In aortic regurgitation, though there is but little obstruction to be overcome, the hypertrophy is sometimes so massive as to acquire the name "Cor Bovinum." This hypertrophy is caused by no obstruction to the outflow, but is evoked by the regurgitant current of blood, which has a far greater distending power than normal, since it is driven backward on the aortic recoil through the imperfect valves, there to meet the current already pouring in through the mitral ostium. This wedge-like distending force causes the heart fibres to stretch, and some dilatation is always found even with this massive hypertrophy.

In reminding you of the real reason why the left ventricle hypertrophies in mitral regurgitation, we have another proof that hypertrophy and dilatation are related to increased internal pressure. Here there is no obstruction to be overcome, but there is an increased distending force to be withstood. The left ventricle first becomes dilated from having blood under abnormally high pressure, and increased in quantity, driven into it during its diastolic by the enlarged left auricle. The ventricle then hypertrophies in order to dispose of this extra quantity of blood, partly forward into the aorto, and partly backward into the left auricle. Not alone in the question of diagnosis, but of prognosis and treatment, we should bear in mind that where there is an obstruction to be oercome, as in aortic stenosis, there is pure hypertrophy, usually without dilatation, while with an increased distending force, as in aortic regurgitation, dilatation is never absent, but always accompanies the hypertrophy.

Passing over the physical signs of hypertrophy, which are familiar to you all, I venture the opinion that no symptom, either subjective or objective, more clearly points to enlargement of the ventricles, left and right, than does accentuation of the aortic and pulmonic second sounds, respectively. This accentuation is due to increased arterial tension, brought about by the enlarged ventricle contracting more powerfully, which leads to an increased arterial recoil, with an earlier and more forcible closure of the semi-lunar valves.

A few important considerations to be remembered are the following: In hypertrophy, palpitation, which is the outward visible sign of internal incompetence, is always the evidence that the hypertrophy is insufficient—not that it is excessive.

The simply dilated heart is unrhythmical, and this is increased by any exertion, which results, not in palpitation, for the heart is not equal to this, but in distinct pauses, as the evidence of great enfeeblement. In pure hypertrophy, there is no palpitation. In hypertrophy and dilatation, the patient finds himself out of breath on any effort, and palpitation is easily induced, hence effort is a useful factor in diagnosis and prognosis. Since the amount of urine passed is the measure of the blood pressure in the arteries, and thus a measure of the state of the heart, a fall in bulk suggests that an hypertrophied heart is failing. Failure of the right side, with tricuspid leakage, is a condition that can never be compensated, since there is no muscle tissue behind the lesion to hypertrophy.

While it is true that hypertrophy is not ordinarily a disease, to be combated, but rather a conservative process to be encouraged—nature's check to the dilating process, it is also true that it may, within itself, become destructive, for, when the obstruction which causes it, lies in arteriole contraction, then the ventricular hypertrophy keeps up high pressure within the arteries, and this oerdistention leads to atheromatous changes in them.

Just a word as to treatment. Rest and nutrition are essential to growth and power. Maintain the blood supply to the coronary vessels, and the heart muscle is nourished. Reduce the demands upon the heart by quietude—physiological rest. Increase the energy of the ventricular contractions by Digitalis, thus more perfectly filling the arteries, including those of the heart itself, affording it more nourishment and prolonging its period of diastolic rest. Improve the general nutrition with good food and iron, and by all of these means you will encourage muscular growth, and add to the heart's power.

In simple dilatation, the measures just enumerated are still more indicated, rest being of prime importance, and a few days in bed now and then, will often enable a failing heart to recover itself.

In pure hypertrophy, or with perfect compensation, Digitalis is not generally indicated, unless it be in very small doses, just to *maintain* compensation.

In hypertrophy and dilatation larger doses are required, while in the dilated and failing heart enormous doses are necessary to life; and we must remember that the dose which seems sufficient while the patient is quiet in bed, is not enough when the heart is taxed by the demands of labor.

931 Candler Building.

